# Dynamic-Weight Fusion of 3D Pose Guidance and Image-Based Visual Servoing for Pre-Contact Alignment in Marine Engineering Equipment Assembly

**DOI:** 10.3390/s26196049

**Published:** 2026-09-24

**Authors:** Wenxing Sun, Duanjiao Li, Junwen Yao, Yanjun Ma, Yun Chen, Qi Xiao, Qiang Yu, Yongfei Ma, Yupeng Zou

**Affiliations:** 1 Guangdong Power Grid Co., Ltd., Guangzhou 510030, China; sunwenxing@dky.gd.csg.cn (W.S.); mayanjun@gd.csg.cn (Y.M.); 2 Electric Power Research Institute of Guangdong Power Grid Co., Ltd., Guangzhou 510030, China; liduanjiao@gd.csg.cn (D.L.); yaojunwen@gd.csg.cn (J.Y.); chenyun@gd.csg.cn (Y.C.); 3 Shandong Key Laboratory of Design and Manufacturing for High-End Offshore Oil and Gas Equipment, College of Mechanical and Electronic Engineering, China University of Petroleum (East China), Qingdao 266580, China; by2215020219@s.upc.edu.cn (Q.X.); z24040066@s.upc.edu.cn (Q.Y.); z25040097@s.upc.edu.cn (Y.M.); 4 Engineering Research Center of the Ministry of Education for New Equipment and Technology in Petroleum and Petrochemical Industry, Qingdao 266580, China

**Keywords:** marine engineering equipment, robotic assembly, RGB-D vision, 3D pose guidance, image-based visual servoing, dynamic weighting, small-clearance insertion

## Abstract

The small-clearance assembly of marine engineering equipment requires the robot to correct coupled pose deviations before contact while retaining fine local alignment. This paper presents an eye-in-hand red, green, and blue plus depth (RGB-D) controller that computes a 3D pose-guidance velocity and an image-based visual-servoing (IBVS) velocity in parallel. Distance and attitude-angle errors set a bounded gain on the 3D branch, while the IBVS gain remains fixed; both velocities are expressed in the same six-dimensional end-effector frame. Each of three initial-deviation conditions was repeated five times for IBVS, 3D Pose Guidance, and Dynamic Fusion. With convergence defined as a mean 3D feature error below 0.3 mm for 30 consecutive frames, Dynamic Fusion reached the threshold and ended within it in all 15 clean trials. IBVS ended within the threshold in 14 of 15 trials and 3D Pose Guidance in 12 of 15. Under combined deviation, Dynamic Fusion converged in 17.73±1.43 s, compared with 24.14±4.81 s for IBVS, and had a terminal error of 0.214±0.011 mm. With injected camera–end-effector calibration perturbations, Dynamic Fusion retained 10/10 terminal successes, whereas 3D Pose Guidance retained 7/10. A single terrestrial physical demonstration showed visual alignment followed by successful insertion; it is treated as a feasibility observation rather than a success-rate evaluation. An additional five-trial fixed-gain ablation converged in 10.17±0.34 s with 0.127±0.040 mm terminal error under clean combined deviation, outperforming the recorded dynamic-fusion results in that condition. The evidence supports feasibility within the tested conditions, but it does not establish the superiority of dynamic weighting over fixed-gain fusion.

## 1. Introduction

Marine engineering equipment—including connectors and sensor housings for seafloor observatory networks, modular interfaces for marine energy systems, and valve and joint assemblies for subsea manifolds—often requires high-precision mating and insertion during manufacturing [1]. Some of these components have submillimeter fit clearances, making pre-contact pose alignment an important factor in first-pass assembly success and subsequent reliability. The broader adoption of industrial and collaborative robots has increased interest in automating such precision assembly operations [2,3,4].

Service conditions place strict limits on manufacturing error. Connector assemblies must remain sealed in high-pressure, corrosive seawater; modular interfaces may be blindly mated underwater by a remotely operated vehicle (ROV) or autonomous underwater vehicle (AUV); and sensor housings require the alignment of optical windows and electrical contacts. Assembly errors beyond the relevant interface tolerances may compromise sealing, electrical continuity, or mechanical engagement during operation. The robot must therefore achieve a small terminal error while moving stably from a relatively large initial deviation into the insertable region.

Figure 1 shows the broader marine engineering context considered here, including representative subsea equipment and ROV-based intervention. Many such operations require a tool or mating component to reach a constrained relative pose before contact. The corresponding visual controller must handle relatively large initial deviations and still provide stable correction near the desired pose.

Robotic assembly has moved from fixed-trajectory execution toward perception-driven closed-loop control. Studies of compliant assembly and force control [5,6,7] showed that small-clearance tasks can be highly sensitive to pre-contact alignment errors because small positional or angular deviations may generate substantial contact forces. Recent reviews [8,9,10,11] also describe the integration of visual perception, force/torque feedback, and compliant control in high-precision assembly. RGB-D cameras are a common sensing choice in such systems because a single device provides both dense depth and aligned color, which supports target localization and image-plane feedback from one observation. This paper focuses on pre-contact visual alignment, where the end-effector pose error is reduced to a safe insertion envelope before physical contact.

Three-dimensional visual pose estimation provides the spatial position and attitude of the target relative to the end effector and is therefore suitable for coarse positioning under large initial deviations. Methods ranging from the classical iterative closest point (ICP) algorithm [12] and generalized ICP [13] to learning-based approaches such as Deep Closest Point [14], RPM-Net [15], CoFiNet [16], and GeoTransformer [17] can estimate the rigid transformation between sensed and model point sets. Near the assembly target, however, depth-sensor noise, sparse-feature reconstruction error, and hand–eye calibration residuals are directly reflected in the end-effector velocity command and may cause low-amplitude oscillation around the target. A controller relying solely on 3D pose guidance can approach rapidly, but its terminal stability may deteriorate.

Image-based visual servoing (IBVS) takes a different approach. It corrects end-effector motion directly from image-plane feature error without recovering a complete 3D pose [18,19,20,21,22]. When the features remain visible and the initial error lies within the local convergence region, IBVS can provide sensitive local correction. Its effective range is limited by feature visibility and by the local approximation in the image interaction matrix. If the target is far from its desired image location or the initial attitude error is large, IBVS alone may converge slowly or lose features [23]. These characteristics favor 3D pose guidance during target approach and a greater role for IBVS near the desired pose.

Relevant work includes staged visual servoing and multimodal assembly control. Chang [24] used an eye-in-hand visual-servoing scheme for smartphone back-shell assembly, dividing the task into long-range approach and short-range assembly. Ahn et al. [25] assigned visual and force information to separate modules in a reinforcement-learning strategy for peg-in-hole assembly. The CFVS framework [26], EA-CTFVS framework [27], and multistage visual-servoing studies [19] further support coarse-to-fine control for six-degree-of-freedom assembly alignment. Here, 3D pose guidance and IBVS are fused continuously in one velocity loop, with error-dependent weighting linking spatial approach to local fine alignment.

Common ways to combine complementary visual-control laws include staged switching and fixed-weight fusion. Staged methods join different control laws at preset thresholds and may be sensitive to threshold selection or exhibit switching transients. Fixed weights avoid discrete switching but do not adapt to the changing error scale during approach. Small-clearance insertion needs strong spatial guidance at first, while the influence of residual 3D-estimation error should diminish near the target. The proposed controller therefore expresses both velocities in the same end-effector space and updates the 3D-branch weight from distance and attitude-angle errors. Table 1 summarizes its relationship to representative coarse-to-fine strategies.

From a sensing perspective, the two error branches are driven by one RGB-D observation stream. The same registered depth image supplies the back-projected sparse 3D points and the depth values used in the IBVS interaction matrix, so feature visibility and depth validity directly gate the control update: the controller checks the completeness of correspondences and depth measurements in every cycle and suspends the update when the observation is invalid. Jointly using the color and depth channels of a single sensor frame is what allows the complementary 3D and image-plane error signals to be generated consistently within the same feedback loop. This sensing structure, together with the error-driven weighting, forms the basis of the proposed fusion controller.

The principal contributions of this paper are as follows:

1. A dual-error feedback model uses corresponding image features and depth measurements to compute 3D pose error and image-feature error from the same RGB-D observations.
2. The 3D pose-guided and IBVS velocities are fused in parallel in a unified six-dimensional end-effector velocity space, enabling spatial approach and local correction within one continuous control loop.
3. A bounded gain driven by distance and attitude-angle errors retains spatial guidance during approach and progressively reduces the contribution of the 3D branch near the target. Repeated simulations quantify terminal accuracy, convergence, computation time, and sensitivity to measurement and calibration perturbations.

The remainder of this paper is organized as follows. Section 2 introduces the eye-in-hand visual assembly system, the equivalent small-clearance assembly task, and the formal statement of the pre-contact coarse/fine alignment problem. Section 3 establishes the visual error model. Section 4 details the dynamic-weight fusion method. Section 5 reports experimental results in terms of approach from large initial deviations, terminal fine-alignment performance, and the dynamic-weight transition. Section 6 concludes the paper and discusses limitations and future work.

## 2. System Description and Equivalent Assembly Task

### 2.1. Eye-in-Hand Visual Assembly System

Visual errors are mapped to robot motion through the transformations among the camera, end-effector, and target frames. Forward kinematics gives the end-effector pose in the base frame, hand–eye calibration gives the fixed camera-to-end-effector transformation, and the vision module estimates the target state in the camera frame. Before fusion, both velocity branches are transformed to the end-effector frame through the adjoint matrix. This mapping includes axis rotation and the linear–angular coupling caused by the camera offset.

Because the camera moves with the end effector, target position, scale, and perspective change in the image. At shorter observation distances, small end-effector motions produce measurable image displacement, while depth noise and occlusion may have greater influence. The controller therefore computes separate velocity terms from 3D pose error and 2D image error and adjusts their relative effects according to the current state.

Visual acquisition, error computation, and robot execution together form a discrete feedback loop. Each image frame undergoes feature detection, depth retrieval, and pose estimation to produce an end-effector velocity. At the execution side, the six-dimensional velocity is integrated into a pose increment according to the measured feedback period after which inverse kinematics generates the joint command. A fixed computation sequence, cycle timestamps, and velocity limits jointly constrain the motion increment in each cycle. All three methods compared in the experiments use the same vision and execution pipeline.

The experimental system adopts the eye-in-hand configuration shown in Figure 2. An Intel RealSense D435 RGB-D camera is rigidly mounted near the end effector of an AUBO-i5 six-degree-of-freedom collaborative robot. The camera moves with the end effector and continuously observes the target workpiece on the table. The RGB channel detects fiducial visual features, while the registered depth channel supplies the corresponding depth values for 3D reconstruction. Four coordinate frames are involved: the robot base frame {B}, end-flange frame {E}, camera frame {C}, and target-workpiece frame {O}. Their transformations are annotated in Figure 2. The square peg tool is fixed to the robot end effector, and the target workpiece is placed on the worktable.

Figure 2 summarizes the sensing and control loop. Target features and their registered depths provide both the 3D spatial deviation and the 2D image-feature error. Dynamic weighting combines the two velocity terms, after which translational and rotational limits are applied before robot execution. The next observation measures the resulting relative-pose change.

### 2.2. Equivalent Small-Clearance Assembly Task

The square-peg–square-hole task reproduces the lateral and rotational constraints relevant to the pre-contact alignment problem considered here. Its square cross-section is sensitive to lateral offset and rotation about the insertion axis, while the small clearance restricts the permissible residual error. The hole constrains two lateral translational degrees of freedom and rotation about the insertion axis, producing coupled alignment without curved-surface contact.

Geometrically, insertability is not determined solely by the scalar magnitude of position error. If a substantial attitude deviation remains, an edge of the square peg may contact the hole entrance first even when the projected peg center is close to the hole center. Conversely, insertion will also be misaligned if the attitude is nearly correct but the lateral offset exceeds the clearance. The controller must therefore track translational and rotational states simultaneously; neither a single pixel distance nor a single spatial distance is a sufficient criterion for the entire process. This paper uses distance and attitude-angle errors to describe the spatial approach state and then uses multi-feature image error to constrain local end-effector alignment.

A common position-error threshold is used to compare convergence times, and the error must remain stable after entering that threshold. Dimensional tolerances, end-effector mounting error, and material deformation can make the visual error differ from the physical insertion condition, so a subsequent insertion trial provides an additional check of the alignment result.

The target is a 3D-printed workpiece containing a 24mm×24mm square hole. A square peg with a 23mm×23mm cross-section is mounted on the robot end effector, and an AprilTag is positioned above the hole. The theoretical clearance on each side is approximately 0.5mm. The specific convergence criterion, initial deviations, and comparison methods are given in Section 5.

The experiments examine target approach and attitude correction before the square peg contacts the hole entrance, and they use the subsequent physical insertion to verify the pre-contact alignment result.

### 2.3. Pre-Contact Coarse/Fine Alignment Problem

The two errors have different useful ranges. When spatial deviation is large relative to depth-measurement fluctuation, the registered 3D pose provides a useful approach direction. At millimeter-scale error, similar measurement fluctuations have greater relative influence on the velocity command. Image-feature error is useful locally, but the interaction-matrix approximation becomes less accurate far from the desired state. The fusion controller varies the relative effects of the two branches with error scale.

Visibility and motion safety also constrain the controller. A large attitude correction may push target features toward the image boundary, whereas excessive translational velocity may carry the end effector across the local alignment region before the next frame arrives. The controller must retain enough range for the early approach and reduce its command near the target. Section 4 addresses these requirements through dynamic weighting and componentwise saturation: the weight allocates control action, and saturation limits the commanded motion.

The pre-contact alignment task is formalized as follows. Let a deviation exist between the current end-effector pose and the desired assembly pose, which is decomposed into translational and rotational components. Before contact occurs, the control objective is to use visual feedback alone to drive this deviation within the permissible bounds determined by the physical clearance.

For controller design, pre-contact alignment is treated as a transition from target approach to terminal fine alignment. Spatial pose information guides motion from a relatively large deviation, while image-feature feedback gains relative influence when correcting small residuals near the desired pose. Both terms are allocated within one velocity framework.

## 3. Visual Measurement and Error Modeling

### 3.1. Construction of Sparse 3D Feature Points

Feature-point depth is affected by RGB–depth registration errors, depth holes, and mixed pixels at target boundaries. For each frame, the vision pipeline verifies feature correspondences and depth validity, smooths short-term detection fluctuations, and back-projects the image points using their depths. If the observation is incomplete, the error update is suspended and zero velocity is sent for that cycle. Computation resumes when a complete valid observation becomes available.

Several features with known correspondences provide geometric constraints at limited computational cost. Three non-collinear 3D correspondences determine a unique rigid transformation; with independent localization errors, additional correspondences permit least-squares estimation and can reduce the influence of one noisy point. Known correspondences avoid per-frame nearest-neighbor search and target-surface reconstruction. The available information is nevertheless concentrated in a few features, so occlusion, incorrect matching, or systematic localization bias can degrade pose estimation.

Reference observations are recorded at the desired assembly pose, so the desired image coordinates and 3D point set reflect the actual camera installation. Camera and hand–eye calibration establish the coordinate mappings, while the reference observation defines the target state used to compute visual error.

The controller uses a sparse set of corresponding 3D features rather than registering a complete point cloud in every frame. The vision module establishes fixed correspondences from detection identifiers, descriptor matching, or geometric constraints. For feature *i*, let its pixel coordinates be $\left(u_{i},v_{i}\right)$, its depth be $z_{i}$, and the camera intrinsics be $f_{x}$, $f_{y}$, $c_{x}$, and $c_{y}$. Its camera-frame coordinates follow from the pinhole model:

(1) $$p_{i}={\left[\begin{array}{ccc} \frac{\left(u_{i}-c_{x}\right)z_{i}}{f_{x}} & \frac{\left(v_{i}-c_{y}\right)z_{i}}{f_{y}} & z_{i} \end{array}\right]}^{T}.$$

Let the current 3D feature set be $P=\{p_{i}\}$ and the desired set recorded at the target pose be $P^{*}=\left\{p_{i}^{*}\right\}$. This paper uses the four AprilTag corners, so N=4. Feature identifiers or matches define the correspondences directly. Compared with full point-cloud registration, the sparse construction requires fewer point operations and excludes background points not associated with the selected features.

### 3.2. 3D Pose-Error Estimation

Rigid registration calculates the centroids of the current and desired point sets and forms a cross-covariance matrix from the centered points. Singular-value decomposition yields the least-squares rotation, after which translation is recovered from the centroids. With correspondences predetermined by the vision module, this is an absolute-orientation problem for ordered point sets and requires no nearest-neighbor search.

The registration residual measures consistency with a rigid-body model. A small point-set residual does not exclude systematic error from depth bias or hand–eye calibration. The 3D estimate is therefore used mainly for spatial guidance with image feedback receiving greater relative weight near the target.

Attitude-angle error is the norm of the axis–angle form of the relative rotation. This scalar is the minimum rotation angle from the current to the desired attitude. The rotation axis is retained for angular-velocity calculation; $e_{\theta }$ supplies only the magnitude used for weighting.

The 3D pose-guidance term describes the spatial discrepancy between the current feature state and the desired assembly state. A rotation R∈SO(3) and translation $t\in R^{3}$ are obtained by rigid registration so that the current sparse feature set *P* is aligned with the desired set $P^{*}$ in the least-squares sense:

(2) $$\min_{R,\;t}\sum_{i=1}^{N}{∥p_{i}^{*}-\left(Rp_{i}+t\right)∥}^{2}.$$

For the closed-form solution of Equation (2), the centroids of the current and desired point sets are first computed as

(3) $$\bar{p}=\frac{1}{N}\sum_{i=1}^{N}p_{i},\;{\bar{p}}^{*}=\frac{1}{N}\sum_{i=1}^{N}p_{i}^{*}.$$

The two sets are then centered:

(4) $$q_{i}=p_{i}-\bar{p},\;q_{i}^{*}=p_{i}^{*}-{\bar{p}}^{*}.$$

The cross-covariance matrix is formed from the centered points and decomposed by singular-value decomposition:

(5) $$H=\sum_{i=1}^{N}q_{i}q_{i}^{*T}=U\Sigma V^{T}.$$

To avoid a reflection and ensure det(R)=1, the closed-form rotation and translation are

(6) $$R=V\;\mathrm{diag}\;\left(1,1,\det\;\left(VU^{T}\right)\right)U^{T},\;t={\bar{p}}^{*}-R\bar{p}.$$

For controller design, the spatial error is decomposed into two scalars: distance error $e_{d}$ and attitude-angle error $e_{\theta }$. The distance error is the Euclidean norm of the translation:

(7) $$e_{d}=∥t∥.$$

The attitude-angle error is obtained from the axis–angle representation of *R*. If its parameters are (k,θ), then $e_{\theta }=\left|\theta \right|$. The scalar magnitudes $e_{d}$ and $e_{\theta }$ are used as inputs to the dynamic weight in Section 4; the corresponding vectors are retained for velocity calculation.

The axis–angle vector φ=θk retains the direction of the attitude error. Combining the translation with this vector gives the six-dimensional error of the 3D pose branch:

(8) $$e_{\mathrm{pose}}={\left[\begin{array}{cc} t^{T} & \varphi^{T} \end{array}\right]}^{T}.$$

A block proportional gain maps this camera-frame error to a camera-frame pose-guidance velocity:

(9) vposeC=−diagktI3,krI3epose.

Here, $k_{t}>0$ and $k_{r}>0$ are the translational and rotational gains, respectively, and $I_{3}$ is the 3×3 identity matrix. The distance error $e_{d}$ and attitude-angle error $e_{\theta }$ enter the dynamic-weight calculation, while the complete vectors t and φ determine velocity direction. The velocity sign and coordinate-transformation direction are consistent with the current-to-desired point-set transformation defined in Equation (2).

The translation and axis–angle vectors provide an explicit correction direction during approach. Depth noise, feature-reconstruction error, and hand–eye calibration error also enter the registration solution. Near the target, these residuals can produce small pose–velocity commands even when the true alignment error is limited. The controller therefore reduces the 3D contribution as spatial error decreases.

### 3.3. Image-Feature Error and IBVS Velocity

For one point feature, the interaction matrix depends on its normalized image coordinates and depth and maps six-dimensional camera velocity to image motion. Stacking the feature matrices allows the camera velocity to be estimated from the complete image-feature vector. Subject to the rank and conditioning of this matrix, its pseudoinverse provides a least-squares velocity command.

The IBVS interaction matrix uses valid current depths or their filtered values, while the closed-loop target is defined in the image plane. Depth error affects the interaction matrix and hence the computed velocity, whereas the image-feature error itself is defined directly from current and desired image coordinates. Attenuating the 3D term increases the relative contribution of image feedback during terminal correction.

The numerical condition of the pseudoinverse depends on the feature configuration. If the image features are too tightly clustered, some points nearly coincide, or the target attitude produces severe perspective compression, the interaction matrix can become ill-conditioned and the velocity solution becomes more sensitive to pixel noise. In practice, features should remain well distributed and visible throughout target approach. Extension to more complex components would require an explicit feature-quality assessment and the detection of degenerate configurations.

The image-feature error is formed from normalized image coordinates. For the four target points, let $s={\left[x_{1},y_{1},\ldots ,x_{4},y_{4}\right]}^{T}\in R^{8}$ and let $s^{*}$ be the corresponding vector recorded at the target pose. The image error is

(10) $$e_{s}=s-s^{*}.$$

For an explicit interaction matrix, the pixel coordinates of feature *i* are converted to normalized image coordinates:

(11) $$x_{i}=\frac{u_{i}-c_{x}}{f_{x}},\;y_{i}=\frac{v_{i}-c_{y}}{f_{y}}.$$

When its depth $z_{i}$ is known, the corresponding two-row interaction matrix is

(12) $$L_{i}=\left[\begin{array}{cccccc} -\frac{1}{z_{i}} & 0 & \frac{x_{i}}{z_{i}} & x_{i}y_{i} & -(1+x_{i}^{2}) & y_{i}\\ 0 & -\frac{1}{z_{i}} & \frac{y_{i}}{z_{i}} & 1+y_{i}^{2} & -x_{i}y_{i} & -x_{i} \end{array}\right].$$

Stacking all $L_{i}$ by rows gives $L_{s}$ in Equation (13). The expansion includes image motion induced by both translation and camera rotation. Near the desired pose, provided that the features remain visible, it relates small image displacements to local camera motion.

IBVS relates the image-feature velocity to the camera spatial velocity $v_{c}$ through the interaction matrix $L_{s}\in R^{8\times 6}$:

(13) $$\dot{s}=L_{s}v_{c}.$$

The standard IBVS control law produces a camera-frame velocity:

(14) vibvsC=−λLs+es.

Substituting Equation (14) into Equation (13) and applying a locally constant interaction-matrix approximation near the desired pose gives the local closed-loop image-error dynamics:

(15) $${\dot{e}}_{s}=-\lambda L_{s}L_{s}^{+}e_{s}.$$

When $L_{s}$ has the required rank, $L_{s}L_{s}^{+}$ acts as the identity on the observable error subspace. With the Lyapunov function $V_{s}=\frac{1}{2}e_{s}^{T}e_{s}$,

(16) $${\dot{V}}_{s}=e_{s}^{T}{\dot{e}}_{s}=-\lambda e_{s}^{T}L_{s}L_{s}^{+}e_{s}\leq 0.$$

Under the locally constant interaction-matrix approximation, and assuming valid depths, persistent feature visibility, and the required rank condition, ${\dot{V}}_{s}\leq 0$ indicates non-increasing image-error energy in the observable subspace. This local result does not establish convergence from arbitrary initial poses.

Here, λ>0 is the visual-servo gain and $L_{s}^{+}$ is the Moore–Penrose pseudoinverse. IBVS defines feedback error in the image plane, although depth estimates still enter the velocity calculation through the interaction matrix. Its main limitation here is the finite local range: for a large initial image error, the interaction-matrix approximation may be inaccurate or features may be lost. The next section combines the two velocity outputs in the end-effector frame.

## 4. Dynamic-Weight Fusion for Visual Alignment

### 4.1. Dynamic Fusion-Control Strategy

In a threshold-based coarse-to-fine scheme, the active controller changes at a prescribed error level. If the controllers use different error signals or velocity mappings, this change may introduce a velocity discontinuity. The threshold may also require adjustment when the initial deviation or operating condition changes.

The controller in Figure 3 computes the 3D pose-guidance and IBVS velocities in parallel. Distance and attitude-angle errors set the contribution of the 3D term at each cycle. The pose branch has greater influence at large errors; its weight falls during approach, leaving IBVS to provide most of the terminal correction. Spatial approach and local alignment are thus connected without a discrete switch.

### 4.2. Parallel Velocity-Fusion Model

Parallel fusion requires the two velocity components to have the same physical meaning and coordinate representation. The pose branch outputs a six-dimensional velocity generated from translation and axis-angle errors, whereas the image branch initially outputs a camera velocity. Both are converted to the end-effector frame through the complete adjoint matrix of the camera–end-effector rigid transformation. This transformation accounts for both axis rotation and the linear–angular velocity coupling caused by the camera mounting offset. Once the frames are unified, the translational and rotational components of the two vectors are combined separately.

The fusion law keeps the IBVS gain fixed and applies an error-dependent weight only to the 3D pose branch. Image error contributes throughout the motion, while the 3D term weakens as the spatial error decreases.

The two branch velocities need not always point in exactly the same direction. At large errors, the pose branch normally supplies the principal direction while the image branch corrects projection error. Near the target, attenuation of the pose branch prevents it from dominating the executed velocity even if depth noise causes a small conflict with the image branch. This structure uses error-dependent gain allocation to regulate how inconsistent information affects the final motion.

To provide a continuous transition between the two control actions, both camera-frame branch velocities are transformed to the end-effector frame before fusion. Let TCE=(REC,pEC) map camera-frame coordinates to the end-effector frame, and order each twist as ${\left[v_{\mathrm{lin}}^{T},\omega^{T}\right]}^{T}$. For either branch,

(17) vE=AdETCvC,AdETC=REC[pEC]×REC0REC.

The negative sign in the branch control laws converts the current-to-desired apparent target displacement obtained in the camera frame into the camera motion command. Let the transformed branch velocities be vposeE and vibvsE. The fused end-effector velocity is

(18) vE = wposevposeE+kibvsvibvsE.

The first term in Equation (18) is the dynamically weighted 3D pose-guidance velocity, and the second is the fixed-gain IBVS velocity. The gain λ in Equation (14) sets the branch controller response, whereas $k_{\mathrm{ibvs}}$ scales that branch during fusion. Both remain fixed across the reported conditions. The continuous weight mapping avoids the explicit command jump associated with threshold-based switching, although the sampled velocity can still reflect frame-to-frame measurement variation.

When the end effector is far from the target, $w_{\mathrm{pose}}$ is relatively large and vposeE supplies most of the translational and attitude correction. Near the target, $w_{\mathrm{pose}}$ decreases and vibvsE has greater relative influence. The IBVS gains are held constant, whereas the pose-guidance term is regulated by spatial error under the local visibility and conditioning assumptions stated in Section 3.

In Figure 3, both control branches are driven in parallel by the same RGB-D observation. The 3D pose branch generates guidance velocity from distance and attitude errors, while the IBVS branch generates terminal correction from image-feature error. The dynamic weight acts only on pose guidance. The two velocity components are fused and limited before driving the robot, whose motion changes the relative pose in the next measurement and thereby closes the sampled-data visual feedback loop.

Figure 4 details the computation sequence. Distance and attitude-angle errors determine the pose-guidance weight. The weighted pose velocity and fixed-gain IBVS velocity are then added in the end-effector frame, which is followed by separate translational and rotational limits.

### 4.3. Dynamic Weighting Based on Spatial Error

Distance and attitude-angle errors have different units. The coefficients α and β therefore set not only their relative importance but also the scale conversion between them. Parameter selection should account for the permitted translational and attitude ranges and robot velocity limits, such that the raw weight reaches its upper bound under a typical large deviation and falls substantially upon entry into the local alignment region. If α is too large, the pose branch remains excessively influential near the target; if β is too small, a large attitude deviation may not receive sufficient coarse-adjustment velocity. The parameters were tuned for the experimental error ranges and held constant across all three initial-deviation cases.

The linear mapping gives a monotonic dependence on the two spatial-error measures. With clipping, it contains upper-bound, linear, and lower-bound regions. The coefficients and bounds were selected for the position and attitude ranges of the experiments and then held fixed for all three initial-deviation conditions.

The clipping function is continuous at both bounds, although its derivative changes there. The upper bound constrains the strength of pose guidance at large error; the lower bound preserves a weak spatial constraint near the target. The bounds are tuned with reference to low-error velocity fluctuations and steady error so that the 3D pose branch balances approach efficiency against terminal stability.

Dynamic weighting regulates the participation of 3D pose guidance according to the current assembly state. Distance error $e_{d}$ and attitude-angle error $e_{\theta }$ are selected as inputs: the former measures positional departure from the target and the latter measures the magnitude of the full relative-attitude error. The unconstrained pose-guidance weight is

(19) $$w_{\mathrm{raw}}=\alpha e_{d}+\beta e_{\theta }.$$

Here, α and β are positive coefficients governing the effects of distance and attitude-angle error. If either $e_{d}$ or $e_{\theta }$ is large, $w_{\mathrm{raw}}$ increases and strong 3D pose guidance is retained. As the end-effector approaches the target, both errors decrease, the 3D contribution diminishes, and the control emphasis naturally shifts toward IBVS.

For all reported experiments, $e_{d}$ is expressed in meters and $e_{\theta }$ in radians with $\alpha =0.5\;m^{-1}$ and $\beta =0.5\;{\mathrm{rad}}^{-1}$. The pose-branch gain is bounded to $w_{\min}=0.02$ and $w_{\max}=0.30$, and the fixed IBVS-branch gain is $k_{\mathrm{ibvs}}=0.06$. Thus, the admissible dynamic pose-gain range is [0.02,0.30]. These values were selected once for the prescribed experimental error range and then held constant; an exhaustive hyperparameter sweep was not performed.

To prevent an excessive velocity at large errors and an abrupt withdrawal of pose guidance at small errors, the raw weight is bounded above and below:

(20) $$w_{\mathrm{pose}}=\mathrm{clip}\;\left(w_{\mathrm{raw}},w_{\min},w_{\max}\right).$$

Equivalently, Equation (20) is the piecewise mapping

(21) $$w_{\mathrm{pose}}=\left\{\begin{array}{cc} w_{\min}, & w_{\mathrm{raw}}<w_{\min},\\ w_{\mathrm{raw}}, & w_{\min}\leq w_{\mathrm{raw}}\leq w_{\max},\\ w_{\max}, & w_{\mathrm{raw}}>w_{\max}. \end{array}\right.$$

This form explicitly separates the low-weight, linear-transition, and upper-bound regions. Adjacent regions have the same value at each boundary, so the weight itself is continuous although its rate of change differs across a boundary.

The upper bound limits the maximum contribution of 3D pose guidance, while the lower bound retains a finite contribution in the low-error region. Distance and attitude-angle errors are recomputed during every visual-feedback cycle. The pose-guidance weight is therefore updated online from the measured error state and continuously represents the allocation of control action between the two velocity branches.

### 4.4. Fusion-Control Flow and Velocity Constraints

Translational and rotational velocities are constrained separately because their numerical scales, mechanical risks, and actuation capabilities differ. Excessive translation could carry the end-effector across the safe alignment region within one feedback interval, whereas excessive angular velocity could sweep a tool edge across the hole entrance. Componentwise norm limiting preserves the direction of each translational or rotational velocity vector while bounding its magnitude. This limits command peaks caused by measurement fluctuations but does not replace the invalid-observation checks described below.

The control loop updates the 3D pose and dynamic gain only when feature detection succeeds, correspondences are complete, and depths are valid. If an observation is invalid, the update is suspended and zero velocity is sent for that cycle; closed-loop computation resumes after a complete and valid observation is restored. The same criterion is used online and offline: convergence is latched after the mean 3D feature error remains below 0.3 mm for 30 consecutive control frames.

The same visual frame is supplied synchronously to both error branches. The 3D points are back-projected using the complete pinhole model, and the IBVS interaction matrix uses valid depths from that frame. Weight calculation and velocity fusion then use pose and image errors with the same sampling instant. The actuator receives the limited velocity, and the robot motion produces a new observation in the following cycle.

Feature jitter, depth fluctuation, and registration error can produce instantaneous peaks after velocity fusion. To ensure stable motion in small-clearance assembly, the end-effector’s translational and angular velocities are constrained separately:

(22) $$∥v_{\mathrm{trans}}∥\leq v_{\mathrm{trans},\max},\;∥v_{\mathrm{rot}}∥\leq v_{\mathrm{rot},\max}.$$

To preserve component ratios, each nonzero translation or rotation vector is scaled without changing direction:

(23) $${\hat{v}}_{j}=\left\{\begin{array}{cc} \;0, & ∥v_{j}∥=0,\\ \min\;\left(1,\frac{v_{j,\max}}{∥v_{j}∥}\right)v_{j}, & ∥v_{j}∥>0, \end{array}\right.\;j\in \left\{\mathrm{trans},\mathrm{rot}\right\}.$$

Here, ${\hat{v}}_{j}$ is the limited velocity vector. If the original velocity does not exceed its bound, the scale factor is one; otherwise, only the vector norm is reduced and its direction is unchanged. The quantities $v_{\mathrm{trans},\max}$ and $v_{\mathrm{rot},\max}$ are the permitted maximum translational and angular velocities. Thus, Equation (23) suppresses instantaneous peaks caused by measurement fluctuation without changing the direction of the corresponding translational or rotational component.

In each control cycle, the system first acquires RGB and depth images, extracts target features, and establishes their correspondences. The same features then provide the 3D pose error and image-feature error from which the two velocity components are generated. The dynamic weight is updated from the current spatial error, the velocities are fused according to Equation (18), and translational and rotational limits are applied according to Equation (23). Using the measured cycle duration, the execution module integrates the limited six-dimensional velocity into an end-effector pose increment, obtains joint commands by inverse kinematics, and sends them to the manipulator.

The calculation is repeated at each visual-feedback cycle. As spatial error falls, the gain of the 3D branch decreases and the fixed-gain IBVS term accounts for more of the composite velocity. This change preserves the approach action of the pose branch while reducing its influence during fine alignment.

## 5. Experiments and Results Analysis

### 5.1. Experimental Platforms and Comparison Methods

Visual features, desired state, coordinate transformations, valid-observation criteria, and velocity safety constraints were identical across the three methods. Each controller was initialized independently. IBVS did not use 3D pose guidance, 3D Pose did not use image-based closed-loop correction, and Fusion used both branches while updating the weight online. The three types of initial deviation covered controller behavior under translational correction, attitude correction, and their coupling.

The primary error metric was the mean Euclidean distance between the four current and desired back-projected 3D features. Convergence time was measured from control activation until this error remained below 0.3 mm for 30 consecutive frames. A latched trial could subsequently move outside the threshold because sensing continued while the controller held zero command; the terminal-within-threshold count therefore provides a separate stability check. Terminal errors were calculated as the mean over the final 5 s of valid observations. End-effector velocity, dynamic gain, and per-cycle computation time were also recorded. Means and sample standard deviations are calculated across five independent trials with the same initial condition. Failed and target-loss trials remain in the terminal-error statistics, while convergence-time statistics use only trials that latched convergence and report their count explicitly.

The simulation environment was used to compare the error and velocity responses of the three methods under consistent initial poses and observation conditions. The physical platform introduced sensor noise, execution error, and workpiece tolerances and was used to examine whether the fusion method supported alignment and insertion on the reported apparatus. The two settings therefore provided a controlled process comparison and an apparatus-level insertion test.

Experiments were conducted in a Gazebo-based robotic visual-assembly simulation and on a physical robotic insertion platform. Both used a six-degree-of-freedom manipulator, an Intel RealSense D435 RGB-D camera, a target workpiece carrying an AprilTag, and a square-peg–square-hole assembly pair. The simulation provided controlled initial deviations for comparing error trajectories, whereas the physical platform examined the method with sensor noise, execution error, and workpiece tolerances.

The simulated RGB-D stream had a resolution of 640×480 pixels and an update rate of 30 Hz. The controller used a pinhole model with $f_{x}=604.25$, $f_{y}=603.036$, $c_{x}=324.038$, and $c_{y}=240.541$ pixels. The simulated stream was undistorted, so all five distortion coefficients were zero. The visual callback was scheduled every 30 ms. The commanded velocity was obtained by applying a scale factor of 0.5 after separate translational and rotational saturation. The simulation and timing measurements ran on a computer with an AMD Ryzen 7 5800H CPU, 16 RAM, and an NVIDIA GeForce RTX 3060 Laptop GPU under Ubuntu 22.04, ROS 2 Humble, and Gazebo 11.10.2; the visual-control calculation used the CPU. Table 2 lists the principal camera and experiment parameters.

Figure 5 shows the terrestrial apparatus used for equivalent-task validation. In Figure 5a, the RGB-D camera is fixed near the manipulator end effector, moves together with the square-peg tool, and continuously observes the target workpiece on the bench. This mounting arrangement is consistent with the eye-in-hand configuration in Figure 2. Figure 5b shows the relative positions of the square peg, square-hole target, and AprilTag. The tag provides stable corner features and depth-sampling locations, while the peg and hole form the actual alignment pair.

Before each trial, the manipulator returned to the prescribed initial joint configuration, and the reference observation averaged from 100 frames at the desired pose was loaded. Each method–condition pair was repeated five times after an independent reset. The position condition applied a local translation of (0,40,30) mm, the angular condition applied a ${\left(0,10,0\right)}^{\circ }$ local rotation, and the combined condition applied both. The three controllers used the same sensing, execution, velocity-limit, reference, and initial-condition settings. RGB images and registered depth images were acquired from the same simulated RGB-D sensor. Errors and velocities were updated only when all four AprilTag correspondences and their depths were valid; otherwise, zero velocity was sent. Each active trial was allowed up to 60 s. Figure 6, Figure 7, Figure 8, Figure 9 and Figure 10 present the repeated simulation results, while Figure 11 retains the previously reported single physical insertion as an apparatus-level feasibility observation.

Three initial-deviation conditions were designed to evaluate the methods under different circumstances:

- **Position deviation:** a translational offset only, which is used to evaluate target-approach capability under a large Cartesian error.
- **Angular deviation:** a rotational offset only, which is used to evaluate attitude-correction performance.
- **Combined pose deviation:** simultaneous translational and rotational offsets, representing a practical assembly situation in which the end effector is displaced and misoriented relative to the target.

The following three control methods were compared:

- **IBVS:** pure image-based visual servoing (Section 3.3).
- **3D Pose:** pure 3D pose-guided control (Section 3.2).
- **Fusion:** the proposed dynamic-weight fusion controller (Section 4).

The metrics comprised the mean 3D feature-error curve, commanded end-effector velocity response, dynamic-gain evolution, terminal error, convergence time, latched-convergence count, terminal-within-threshold count, and target-loss count. Table 3 summarizes the functional roles of the three controllers.

### 5.2. Repeated Baseline Comparison

Figure 6 reports all five repetitions for each controller and initial-deviation condition. Thin lines denote individual trials and thick lines denote their pointwise median. The logarithmic ordinate retains both the large initial error and the 0.3 mm terminal region. All three methods reduced the error in the position- and angular-deviation conditions. The combined condition was more discriminating: Dynamic Fusion reached and retained the terminal region in all five trials, IBVS reached it in all trials but one trial ended above the threshold, and 3D Pose lost the target or valid depth in one trial.

The two success definitions distinguish the transient threshold entry from the terminal state. Dynamic Fusion both latched convergence and ended below 0.3 mm in 15/15 trials. The corresponding terminal counts were 14/15 for IBVS and 12/15 for 3D Pose. Fusion was not the fastest method in every condition: IBVS was faster for the position and angular offsets. Under the more difficult combined offset, however, Fusion was 6.42 s faster on average than IBVS while retaining 5/5 terminal successes. The large mean and standard deviation of the 3D Pose terminal error in that condition result from retaining the target-loss trial rather than removing it from the analysis.

Figure 7 shows that the three methods also differed in command evolution. The 3D Pose branch produced the largest initial command and the greatest between-trial variation. IBVS generated a smaller, longer-lived command. Fusion reduced both linear and angular commands while its 3D gain declined, and its command reached zero after convergence. These data support the intended continuous redistribution of control effort; they do not establish that Fusion is uniformly faster than either single controller. Table 4 reports the repeated baseline results.

### 5.3. Fixed-Gain Fusion Ablation

Five additional clean combined-offset trials used the same fusion implementation with the pose-branch gain fixed at 0.10 and the IBVS gain fixed at 0.06. The pose gain was selected before these trials as one representative constant within the dynamic gain range [0.02,0.30]; it was not selected by optimizing the reported results. The desired features, prescribed initial pose, filtering, saturation, command scale, and convergence criterion were retained. The recorded initial feature errors ranged from 78.421 mm to 78.434 mm. These additional trials were collected in a later session and compared with the five previously recorded dynamic-fusion trials; the comparison was not randomized or interleaved across sessions.

Both methods latched convergence and ended below 0.3 mm in 5/5 trials. Fixed-gain fusion converged in 10.17±0.34 s with a terminal feature error of 0.127±0.040 mm compared with 17.73±1.43 s and 0.214±0.011 mm for dynamic fusion. Thus, this ablation does not demonstrate a performance advantage of dynamic weighting in the tested clean combined-offset condition. A constant pose gain of 0.10 performed well for this initial state. Error-dependent scheduling remains a mechanism for varying the pose contribution, but its benefit relative to a suitable constant gain must not be inferred from the single-controller baselines. Broader gain sweeps and matched comparisons across noise levels and initial conditions are needed to establish where scheduling is beneficial.

### 5.4. Noise, Calibration, and Observation-Loss Robustness

The combined-offset condition was repeated with additive RGB-D measurement noise and camera–end-effector calibration perturbations. The low-noise setting used 1 pixel feature noise and 3 mm depth noise; the high-noise setting used 2 pixel and 6 mm. Calibration perturbations had magnitudes of 5 mm and 1° (low) or 10 mm and 2° (high). These tests compare Fusion with 3D Pose because the perturbations directly probe the RGB-D pose branch that is attenuated by the proposed gain schedule.

Neither controller met the strict 30-frame criterion under continuously injected noise. The noise affects both the feedback command and the measured stopping criterion. At the approximately 0.329 m reference depth and focal length of about 604 pixels, one pixel corresponds to approximately 0.545 mm laterally. Ignoring the smaller off-axis depth coupling, the injected single-feature 3D root-mean-square perturbation is approximately 3.10 mm at the low setting and 6.20 mm at the high setting. These are raw measurement scales; they are not observed terminal errors or ground-truth positioning errors.

The implementation filters all three feature coordinates with an exponential moving average having coefficient γ=0.2. For independent white input noise and a stationary feature, the filtered standard deviation is multiplied by $\sqrt{\gamma /(2-\gamma )}=1/3$. The corresponding approximate single-feature perturbation scales are therefore 1.03 mm and 2.07 mm, which are still above the 0.3 mm threshold. The evaluation averages four Euclidean feature-error norms rather than averaging signed coordinate errors; noise contributions consequently do not cancel directly. Filtering also correlates consecutive samples, so an independence-based probability calculation for the 30-frame rule would be inappropriate. Table 5 reports the perturbation results.

These scale estimates explain a measurement limitation but do not prove that the true robot pose has converged. Perturbed features also change the reconstructed rigid transformation and the IBVS interaction matrix, creating command fluctuations and potentially a residual pose offset. Error-dependent gain scheduling is not a noise estimator: noisy pose errors can keep the 3D gain elevated, its lower bound retains a nonzero pose contribution, and the fixed-gain IBVS branch still receives perturbed features. These mechanisms are consistent with the observed degradation, although the present records do not isolate their individual causal contributions. Fusion had lower mean terminal errors at both noise levels, but neither controller satisfied the specified convergence criterion. Independent ground-truth pose evaluation would be needed to separate actual alignment error from sensing error. IBVS was not rerun in the perturbation block, so these results do not rank all three controllers under noise or calibration error.

Under calibration perturbations, Fusion retained 10/10 terminal successes, whereas 3D Pose retained 7/10. Three 3D Pose trials—one clean and one at each calibration level—lost the target or valid depth for more than 10 s. In all three, the controller issued its first zero command within 23.6 ms to 32.3 ms after the last valid observation, and all commands recorded after 100 ms were zero. Control can resume when a complete valid observation returns; no dedicated occlusion-and-recovery trajectory was performed.

### 5.5. Dynamic-Gain Transition and Execution Time

The recorded trajectory in Figure 10 began near 50 mm translation error and 10° rotation error. The 3D pose gain initially remained near 0.112, decreased continuously as both errors fell, and reached its lower bound of 0.02 after approximately 5.5 s; the IBVS gain remained fixed at 0.06. Thus, the implemented method is specifically an error-dependent 3D gain combined with a fixed IBVS gain. It does not use complementary weights that sum to one.

The mean Fusion iteration time was below the 30 ms schedule, but 14.13% of its measured iterations exceeded that period, and its mean trial-level 95th percentile was 32.32 ms. The implementation should therefore be regarded as soft real time on the stated computer rather than as a hard-deadline controller.

CPU and GPU utilization percentages were not logged by the recording node, so the present timing table does not provide a resource-occupancy profile. Dedicated process-level profiling remains future work.

For *n* matched features, back-projection, centroid calculation, covariance accumulation, and image-error construction scale linearly with *n*. The rigid registration uses an SVD of a 3×3 covariance matrix, and the IBVS branch uses the pseudoinverse of an 8×6 interaction matrix for the four-feature implementation; both matrix dimensions are fixed in the reported system. Consequently, image acquisition and feature detection dominate the practical cycle time more strongly than the fixed-size fusion arithmetic. Table 6 reports the measured end-to-end visual-control iteration time rather than relying only on this asymptotic description.

### 5.6. Single Physical Insertion Feasibility Observation

In the retained physical experiment, the vision-estimated position-error norm after alignment was 0.228 mm, after which the square peg was inserted successfully. This single observation demonstrates compatibility between the pre-contact alignment output and insertion in that trial. It does not provide an insertion success rate, insertion-time distribution, or jamming statistic.

Several practical factors may have contributed to the difference between the simulation and physical results. Hand–eye calibration residuals can introduce a systematic offset between the camera-measured and actual end-effector poses. AprilTag detection was also affected by viewpoint changes, particularly during coarse approach when the tag appeared at an oblique angle. The 3D-printed workpieces had dimensional tolerances of approximately 0.1 mm, and the square-peg mounting introduced an additional angular offset. Their individual effects were not isolated in this paper.

No archived independent hand–eye calibration residual or repeated physical-insertion dataset was available for this revision, so the physical demonstration was excluded from the repeated statistics. Repeated contact-rich trials, broader fixed-gain comparisons and a hard-switch ablation, an independent calibration-residual report, and dedicated occlusion recovery remain necessary to quantify task-level reliability beyond the pre-contact simulation study in this paper.

## 6. Conclusions

This paper presented a dynamic-weight fusion method for pre-contact alignment in the precision assembly of marine engineering equipment. It computes 3D pose-guidance and IBVS velocities in parallel in a common six-dimensional end-effector space. Distance and attitude-angle errors set the weight of the 3D branch online. The pose branch remains influential at large deviations and recedes during approach, allowing image feedback to govern terminal fine alignment.

Across 45 clean simulation trials, Dynamic Fusion latched convergence and ended below 0.3 mm in 15/15 trials compared with terminal counts of 14/15 for IBVS and 12/15 for 3D Pose. Under combined deviation, Fusion converged in 17.73±1.43 s with a terminal error of 0.214±0.011 mm. It was faster than IBVS in this condition but not in the position-only or angular-only conditions. Under two camera–end-effector calibration perturbations, Fusion retained 10/10 terminal successes, while 3D Pose retained 7/10. Continuous measurement noise prevented either tested method from satisfying the strict 30-frame threshold, although Fusion yielded lower mean terminal error at both noise levels.

Operation requires complete feature correspondences, valid depth measurements, and a well-conditioned interaction matrix. If the observation is incomplete or contains invalid depth, the controller sends zero velocity for that cycle and resumes computation when a valid observation becomes available. Given valid observations, the dynamic weight regulates the gain of the 3D pose branch.

The evaluation used a terrestrial square-peg–square-hole task with AprilTag correspondences. The retained physical trial showed successful insertion after a measured 0.228 mm pre-contact error, but it cannot establish insertion reliability. The added clean combined-offset ablation favored the tested constant pose gain over dynamic weighting in both mean convergence time and mean terminal error. Thus, the current evidence does not establish a dynamic-weight advantage over fixed-gain fusion. Further work will use repeated physical insertions, broader fixed-gain sweeps and switching ablations, independent calibration assessment, and deliberate occlusion tests. Component-specific geometry and non-fiducial features under underwater optical disturbances, together with force/torque feedback and compliant control, are also needed for contact-rich insertion.

## Figures and Tables

**Figure 1:**
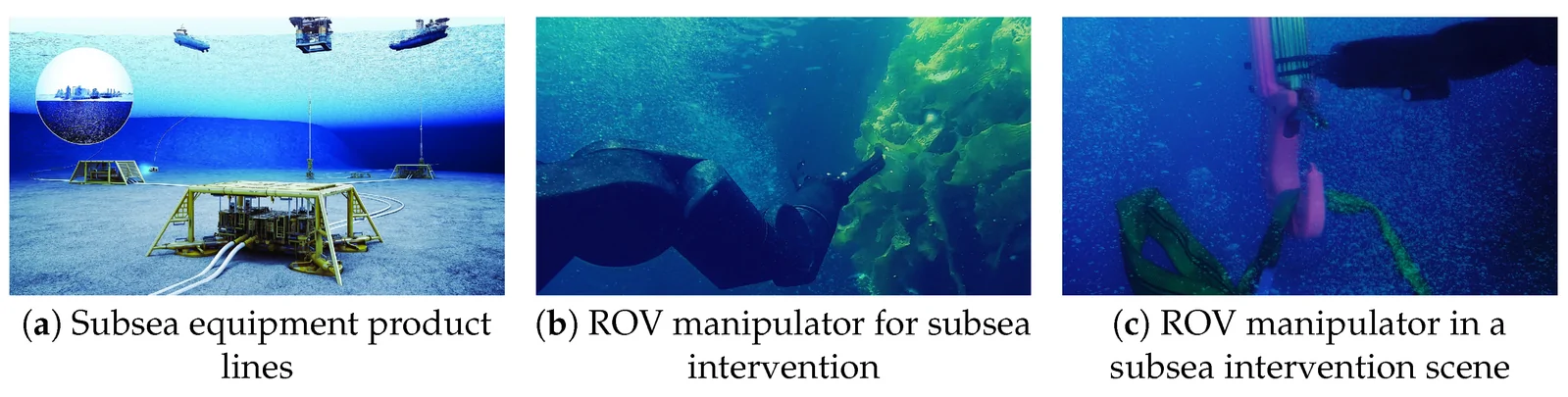
Marine engineering context for precision alignment and assembly: (**a**) representative subsea equipment product lines; (**b**,**c**) remotely operated vehicle (ROV) manipulators used for subsea inspection and intervention. The method is evaluated on the terrestrial small-clearance task defined in Section 2 with the experimental procedure reported in Section 5. **Image credits:** (**a**) FMC Technologies, CC BY 3.0, via Wikimedia Commons; (**b**,**c**) BPLINFO/Blueprint Lab, CC BY-SA 4.0, via Wikimedia Commons. All images were cropped for layout.

**Figure 2:**
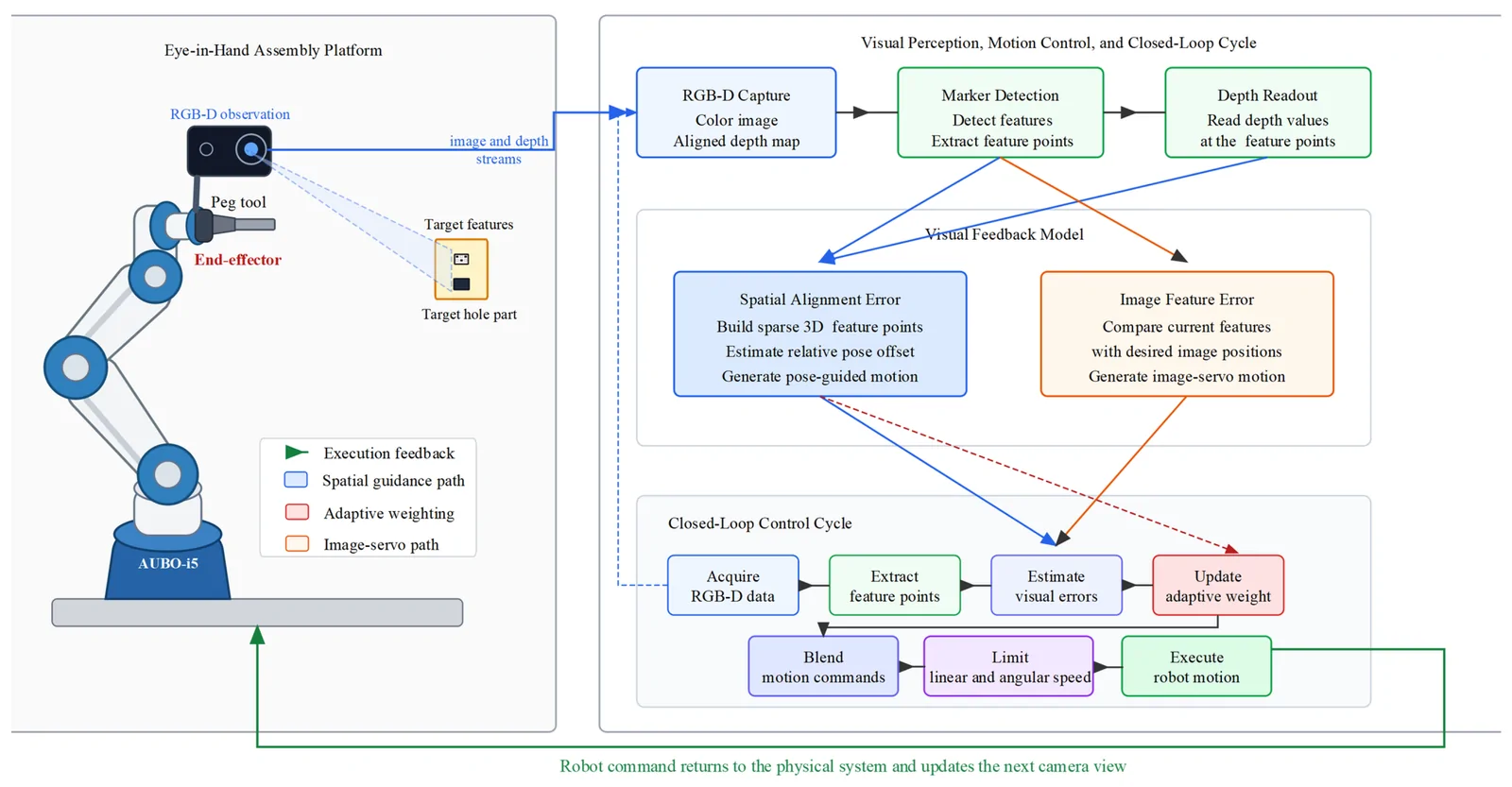
Overall framework of the eye-in-hand visual closed-loop assembly system. **Source:** Author’s own work.

**Figure 3:**
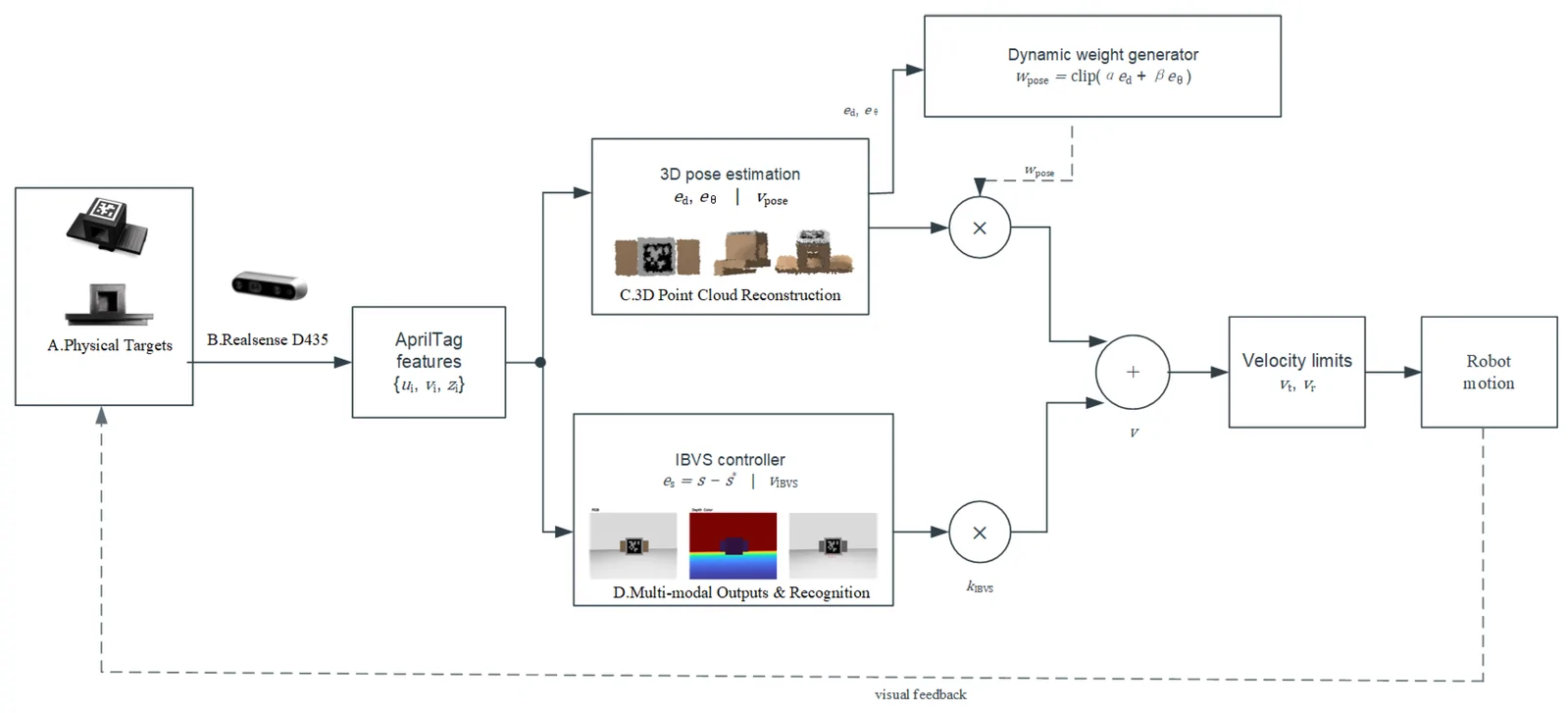
Dynamic-weight fusion framework for visual alignment control. **Source:** Author’s own work.

**Figure 4:**
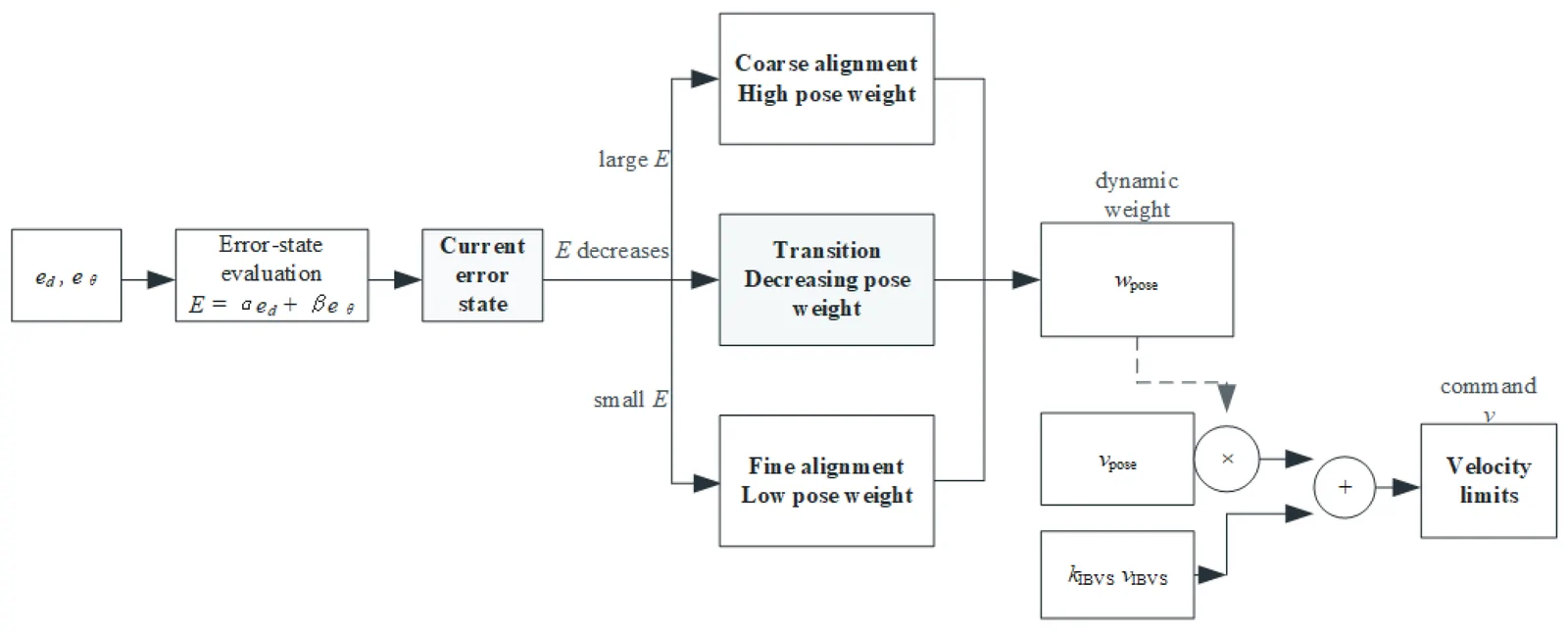
Dynamic-weight fusion-control flow. **Source:** Author’s own work.

**Figure 5:**
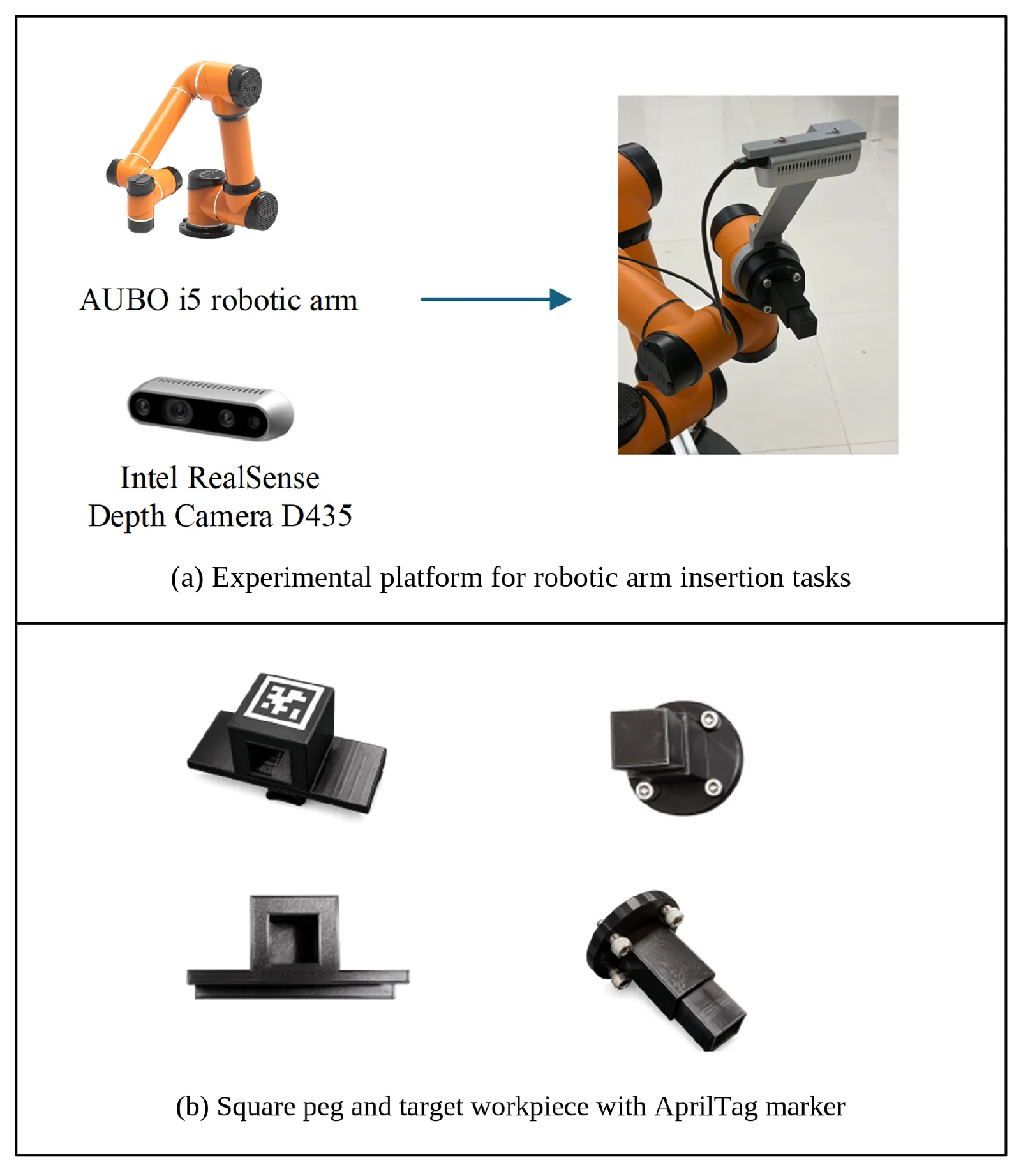
Robotic insertion platform and square-peg–square-hole workpieces: (**a**) AUBO-i5 insertion platform; (**b**) square peg, target workpiece, and AprilTag. **Source:** Author’s own work.

**Figure 6:**
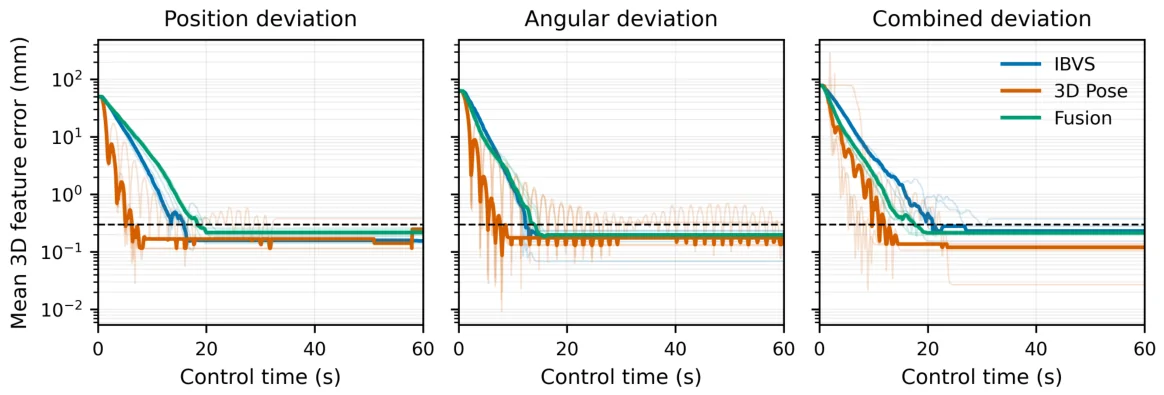
Mean 3D feature-error histories for five repetitions under each initial-deviation condition. Thin lines are individual trials, thick lines are pointwise medians, and the dashed line is the 0.3 mm convergence threshold. Failed trials are retained. **Source:** Author’s own work.

**Figure 7:**
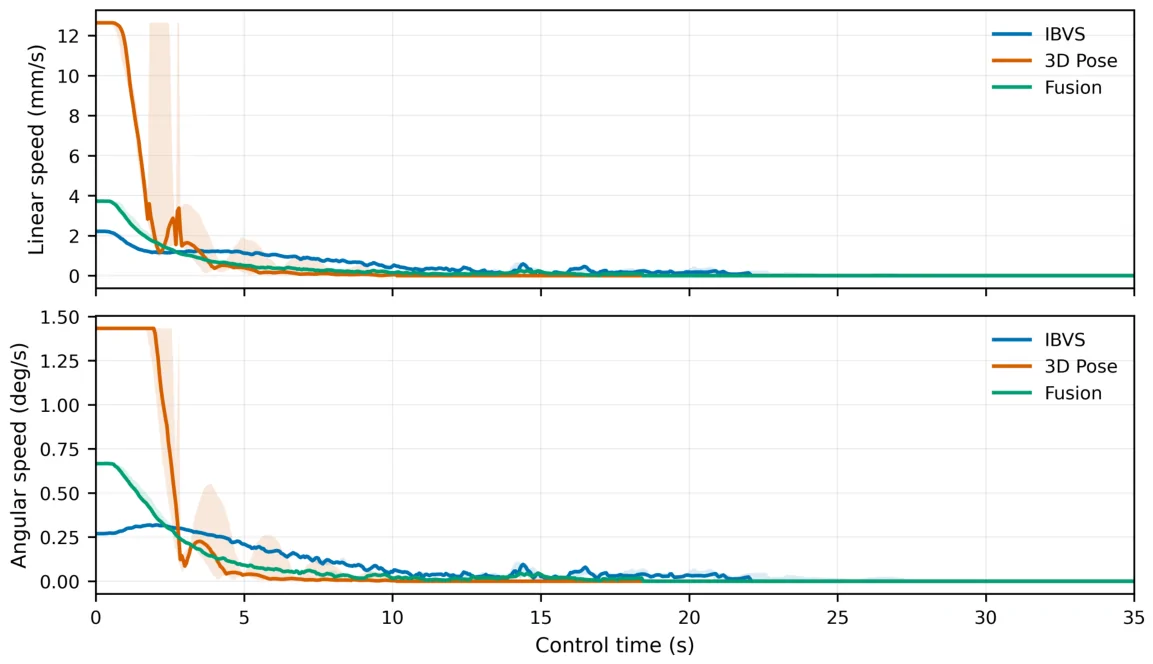
Commanded end-effector speed under combined pose deviation. Solid lines are pointwise medians and shaded regions span the interquartile range of the five repetitions. **Source:** Author’s own work.

**Figure 8:**
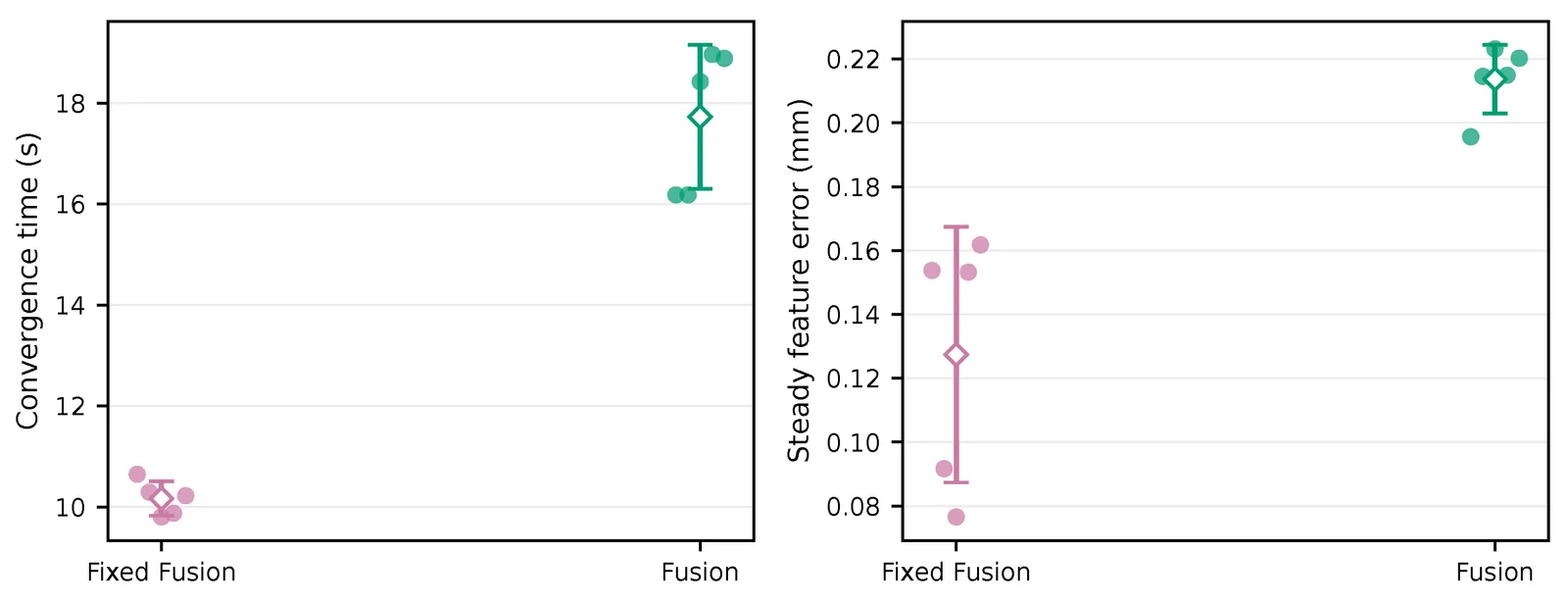
Fixed-gain and dynamic fusion under clean combined deviation (n=5 per method). Points represent individual trials; diamonds and error bars represent mean and sample standard deviation. All ten trials satisfied the convergence criterion. Dynamic-fusion data are from the earlier experiment session. **Source:** Author’s own work.

**Figure 9:**
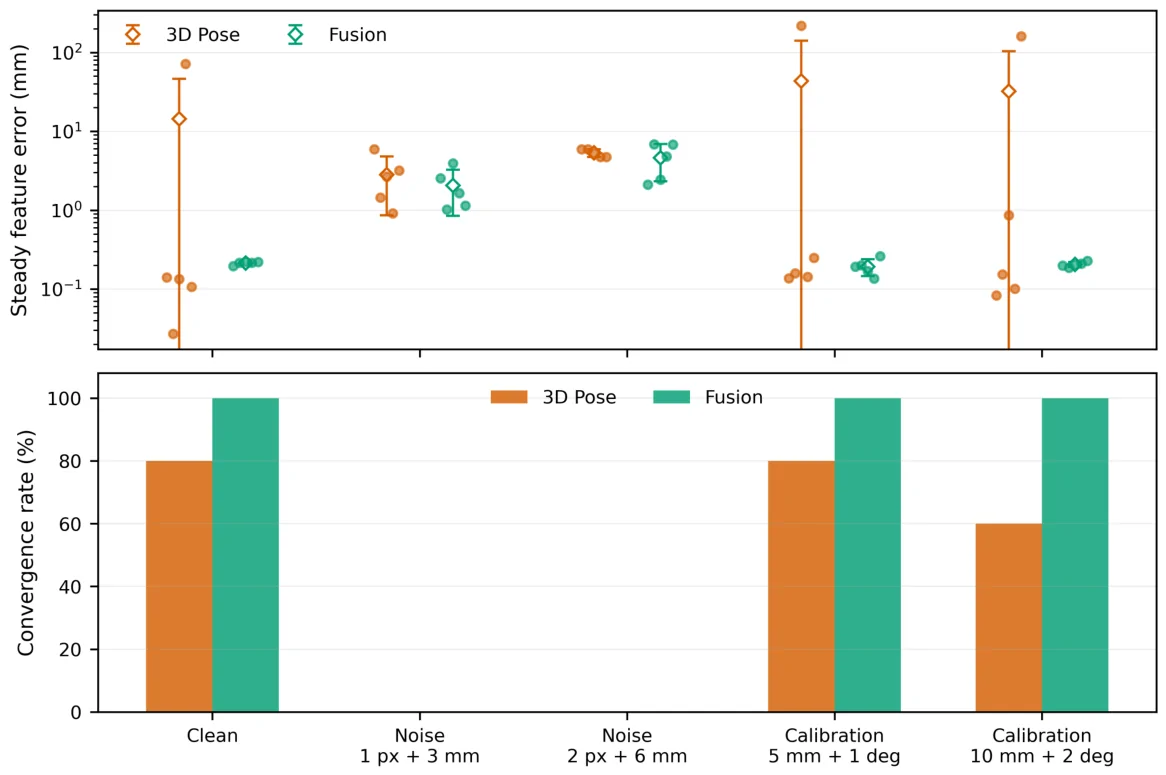
Robustness under combined deviation. Points show individual final 5 s errors, diamonds and bars show mean ± sample standard deviation, and the lower panel gives the strict 0.3 mm/30-frame convergence rate. Failed trials are retained. **Source:** Author’s own work.

**Figure 10:**
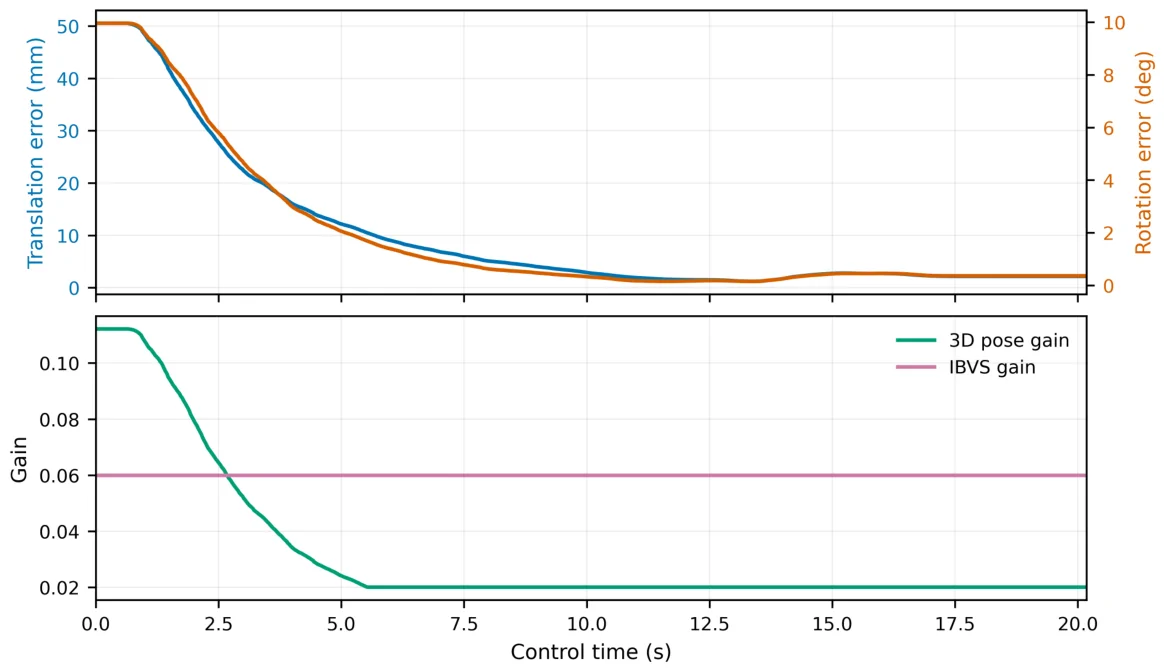
Translation error, rotation error, and gain evolution for a representative combined-offset Fusion trial. The 3D pose gain is error dependent and the IBVS gain remains fixed. **Source:** Author’s own work.

**Figure 11:**
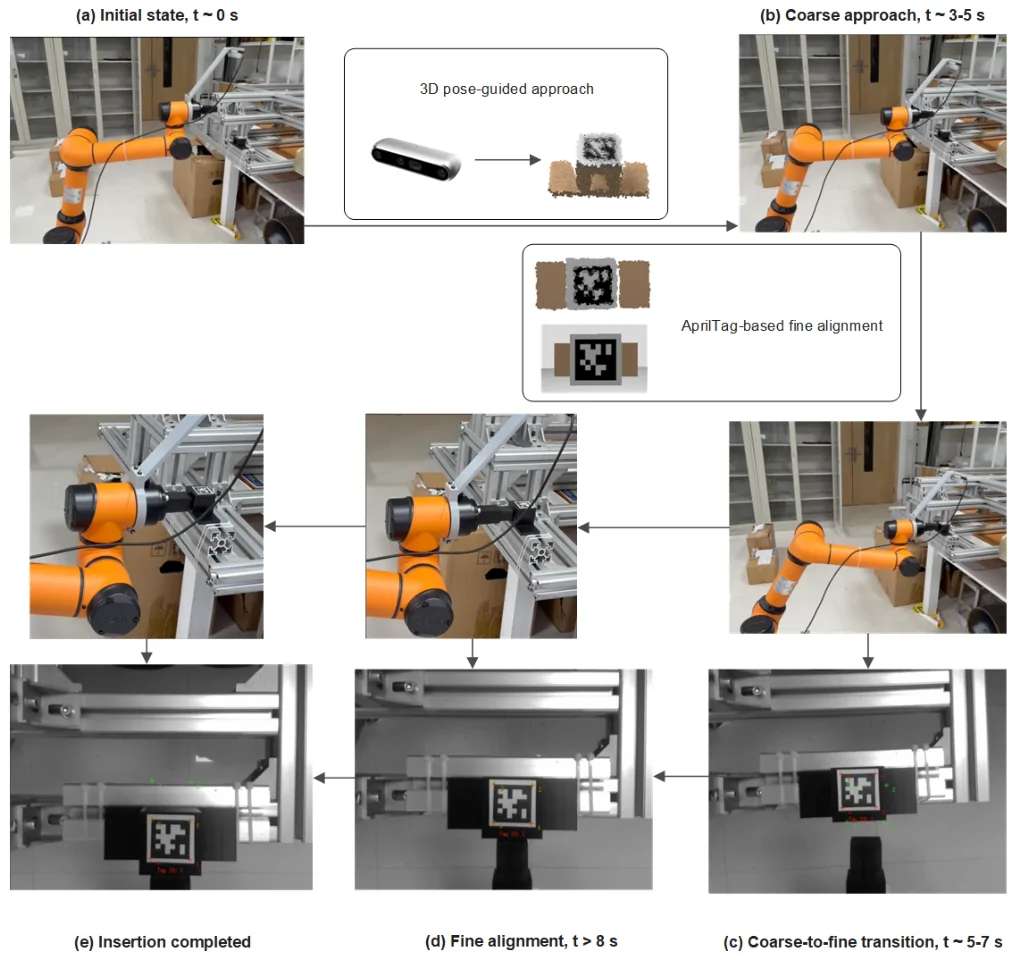
Single physical insertion sequence under Fusion control: (**a**) initial state; (**b**) coarse approach; (**c**) gain transition; (**d**) fine alignment; and (**e**) completed insertion. This demonstration is not a repeated success-rate experiment. **Source:** Author’s own work.

**Table 1:** Positioning of the proposed controller relative to representative coarse-to-fine assembly strategies. **Source:** Author’s own work.

Strategy	Information Allocation	Transition Mechanism	Relation to This Paper
Multistage visual servoing [19,24,26,27]	Separate coarse/fine stages	Discrete stage or threshold	Related task decomposition
Fixed-weight velocity fusion	Constant branch coefficients	No state dependence	Does not reduce the 3D gain near the target
Visual–force learning [25]	Vision and contact-force modules	Policy dependent	Addresses the contact stage beyond the present scope
Proposed Dynamic Fusion	3D pose guidance plus image-based visual servoing (IBVS)	Continuous error-dependent 3D gain	Pre-contact controller evaluated here

**Table 2:** Camera and experiment parameters used in the repeated simulation. **Source:** Author’s own work.

Parameter	Value
Image resolution and sensor rate	640×480 pixels, 30 Hz
Camera intrinsics $\left(f_{x},f_{y},c_{x},c_{y}\right)$	(604.25,603.036,324.038,240.541) pixels
Distortion model	Rectified pinhole; $(k_{1},k_{2},k_{3},p_{1},p_{2})=0$
Visual callback period	30 ms
Control command scale	0.5
Convergence criterion	$e_{3D}<0.3\;mm$ for 30 consecutive frames
Maximum active duration	60 s
Repetitions	5 per method and initial condition
Terminal-statistics window	Final 5 s of valid observations

**Table 3:** Functional comparison of the three control methods. **Source:** Author’s own work.

Method	Control Input	Primary Role
IBVS	Image error	Local correction
3D Pose	Spatial pose error	Coarse guidance
Fusion	Both errors	Continuous fusion

**Table 4:** Repeated baseline results. Terminal error is the mean over the final 5 s of valid observations and is reported across all five trials as mean ± sample standard deviation. Convergence time is calculated only for successful trials; the success count is shown explicitly. **Source:** Author’s own work.

Condition	Method	Latched	Terminal <0.3 mm	Time (s)	Terminal Error (mm)
Position	IBVS	5/5	5/5	17.36±1.69	0.164±0.013
	3D Pose	5/5	4/5	12.41±11.01	0.190±0.110
	Fusion	5/5	5/5	19.70±1.35	0.210±0.046
Angular	IBVS	5/5	5/5	13.24±0.55	0.175±0.069
	3D Pose	5/5	4/5	22.35±22.40	0.178±0.087
	Fusion	5/5	5/5	14.93±0.95	0.193±0.012
Combined	IBVS	5/5	4/5	24.14±4.81	0.250±0.084
	3D Pose	4/5	4/5	14.21±6.35	14.374±31.914
	Fusion	5/5	5/5	17.73±1.43	0.214±0.011

**Table 5:** Robustness results under combined deviation (n=5 per row). Terminal errors include failed trials. **Source:** Author’s own work.

Perturbation	Method	Latched	Terminal <0.3 mm	Terminal Error (mm)
None	3D Pose	4/5	4/5	14.374±31.914
	Fusion	5/5	5/5	0.214±0.011
Noise: 1 px + 3 mm	3D Pose	0/5	0/5	2.829±1.966
	Fusion	0/5	0/5	2.052±1.202
Noise: 2 px + 6 mm	3D Pose	0/5	0/5	5.326±0.593
	Fusion	0/5	0/5	4.604±2.284
Calibration: 5 mm + 1 deg	3D Pose	4/5	4/5	43.566±97.032
	Fusion	5/5	5/5	0.192±0.046
Calibration: 10 mm + 2 deg	3D Pose	3/5	3/5	32.197±71.323
	Fusion	5/5	5/5	0.206±0.015

**Table 6:** Visual-control iteration time over the 15 clean trials per method. The reported overrun rate is the fraction of measured iterations exceeding the scheduled 30 ms callback period. **Source:** Author’s own work.

Method	Mean ± Between-Trial SD (ms)	Mean Trial 95th Percentile (ms)	>30 ms (%)
IBVS	23.30±3.73	35.56	24.06
3D Pose	22.91±3.61	34.64	19.09
Fusion	21.62±2.37	32.32	14.13

## Data Availability

The ROS 2 bag files and analysis code supporting the reported simulation results are available from the corresponding author upon reasonable request. The summary statistics needed to interpret the results are included in the article.

## References

[1] Smith L.M., Barth J.A., Kelley D.S., Plueddemann A., Rodero I., Ulses G.A., Vardaro M.F., Weller R. (2018). The Ocean Observatories Initiative. Oceanography.

[2] Müller C. (2025). World Robotics 2025: Industrial Robots.

[3] Keshvarparast A., Battini D., Battaia O., Pirayesh A. (2024). Collaborative robots in manufacturing and assembly systems: Literature review and future research agenda. J. Intell. Manuf..

[4] Jiang Y., Huang Z., Yang B., Yang W. (2022). A review of robotic assembly strategies for the full operation procedure. Robot. Comput.-Integr. Manuf..

[5] Hogan N. Impedance control: An approach to manipulation. Proceedings of the American Control Conference.

[6] Mason M.T. (1981). Compliance and force control for computer controlled manipulators. IEEE Trans. Syst. Man Cybern..

[7] Whitney D.E. (1982). Quasi-static assembly of compliantly supported rigid parts. J. Dyn. Syst. Meas. Control.

[8] Ji S., Lee S., Yoo S., Suh I., Kwon I., Park F.C., Lee S., Kim H. (2021). Learning-based automation of robotic assembly for smart manufacturing. Proc. IEEE.

[9] Wang H., Salunkhe O., Quadrini W., Lämkull D., Ore F., Despeisse M., Fumagalli L., Stahre J., Johansson B. (2024). A systematic literature review of computer vision applications in robotized wire harness assembly. Adv. Eng. Inform..

[10] Li R., Qiao H. (2019). A survey of methods and strategies for high-precision robotic grasping and assembly tasks. IEEE/ASME Trans. Mechatron..

[11] Suomalainen M., Karayiannidis Y., Kyrki V. (2022). A survey of robot manipulation in contact. Robot. Auton. Syst..

[12] Besl P.J., McKay N.D. (1992). A method for registration of 3-D shapes. IEEE Trans. Pattern Anal. Mach. Intell..

[13] Segal A.V., Haehnel D., Thrun S. Generalized-ICP. Proceedings of the Robotics: Science and Systems.

[14] Wang Y., Solomon J.M. (2019). Deep Closest Point: Learning representations for point cloud registration. Proceedings of the IEEE/CVF International Conference on Computer Vision.

[15] Yew Z.J., Lee G.H. (2020). RPM-Net: Robust point matching using learned features. Proceedings of the IEEE/CVF Conference on Computer Vision and Pattern Recognition.

[16] Yu H., Li F., Saleh M., Busam B., Ilic S. (2021). CoFiNet: Reliable coarse-to-fine correspondences for robust point cloud registration. Proceedings of the Advances in Neural Information Processing Systems.

[17] Qin Z., Yu H., Wang C., Guo Y., Peng Y., Ilic S., Hu D., Xu K. (2023). GeoTransformer: Fast and robust point cloud registration with geometric transformer. IEEE Trans. Pattern Anal. Mach. Intell..

[18] Chaumette F., Hutchinson S. (2006). Visual servo control, Part I: Basic approaches. IEEE Robot. Autom. Mag..

[19] Cao C., Zhao D., Wu X., Liu L., Jiao T., Zhao J. (2024). Investigation of multi-stage visual servoing in the context of autonomous assembly. Measurement.

[20] Kmich M., Karmouni H., Harrade I., Daoui A., Sayyouri M. (2022). Image-based visual servoing techniques for robot control. Proceedings of the International Conference on Intelligent Systems and Computer Vision.

[21] Shen Y., Jia Q., Wang R., Huang Z., Chen G. (2023). Learning-based visual servoing for high-precision peg-in-hole assembly. Actuators.

[22] Zhang Y., Zheng Y., Gao F., Li S. (2025). Image-based visual servoing of manipulators with unknown depth: A recurrent neural network approach. IEEE Trans. Neural Netw. Learn. Syst..

[23] Yu Q., Wei W., Wang D., Li Y., Gao Y. (2024). A framework for IBVS using virtual work. Actuators.

[24] Chang W.C. (2018). Robotic assembly of smartphone back shells with eye-in-hand visual servoing. Robot. Comput.-Integr. Manuf..

[25] Ahn K.H., Na M., Song J.B. (2023). Robotic assembly strategy via reinforcement learning based on force and visual information. Robot. Auton. Syst..

[26] Lu B.S., Chen T.I., Lee H.Y., Hsu W.H. (2023). CFVS: Coarse-to-fine visual servoing for 6-DoF object-agnostic peg-in-hole assembly. Proceedings of the IEEE International Conference on Robotics and Automation.

[27] Bai Y., Dong M., Wei S., Yu X. (2024). EA-CTFVS: An environment-agnostic coarse-to-fine visual servoing method for sub-millimeter-accurate assembly. Actuators.

